# Pulmonary and Systemic Hemodynamics following Multielectrode Radiofrequency Catheter Renal Denervation in Acutely Induced Pulmonary Arterial Hypertension in Swine

**DOI:** 10.1155/2021/4248111

**Published:** 2021-11-02

**Authors:** Aleksandr D. Vakhrushev, Heber Ivan Сondori Leonardo, Natalia S. Goncharova, Lev E. Korobchenko, Lubov B. Mitrofanova, Elizaveta M. Andreeva, Elena G. Koshevaya, Dmitry S. Lebedev, Evgeny N. Mikhaylov

**Affiliations:** Almazov National Medical Research Centre, Saint Petersburg, Russia

## Abstract

**Objective:**

We aimed to assess the effects of renal denervation (RDN) on systemic and pulmonary hemodynamics in a swine model of thromboxane A2- (TXA2-) induced pulmonary arterial hypertension (PAH).

**Methods:**

The study protocol comprised two PAH inductions with a target mean pulmonary artery pressure (PAP) of 40 mmHg at baseline and following either the RDN or sham procedure. Ten Landrace pigs underwent the first PAH induction; then, nine animals were randomly allocated in 1 : 1 ratio to RDN or sham procedure; the second PAH induction was performed in eight animals (one animal died of pulmonary embolism during the first PAH induction, and one animal died after RDN). In the RDN group, ablation was performed in all available renal arteries, and balloon inflation within artery branches was performed in controls. An autopsy study of the renal arteries was performed.

**Results:**

At baseline, the target mean PAP was achieved in all animals with 25.0 [20.1; 25.2] mcg of TXA2. The second PAH induction required the same mean TXA2 dose and infusion time. There was no statistically significant difference in the mean PAP at second PAH induction between the groups (39.0 ± 5.3 vs. 39.75 ± 0.5 mmHg, *P* > 0.05). In the RDN group, the second PAH induction resulted in a numerical but insignificant trend toward a decrease in the mean systemic blood pressure and systemic vascular resistance, when compared with the baseline induction (74 ± 18.7 vs. 90.25 ± 28.1 mmHg and 1995.3 ± 494.3 vs. 2433.7 ± 1176.7 *dyn*∗sec∗*cm*^−5^, *P* > 0.05, respectively). No difference in hemodynamic parameters was noted in the sham group between the first and second PAH induction. Autopsy demonstrated artery damage in both groups, but RDN resulted in more severe lesions.

**Conclusions:**

According to our results, RDN does not result in significant acute pulmonary or systemic hemodynamic changes in the TXA2-induced PAH model. The potential chronic effects of RDN on PAH require further research.

## 1. Introduction

Pulmonary arterial hypertension (PAH) is a fatal disease characterized by a progressive increase in pulmonary vascular resistance, leading to right ventricular failure and earlier death. This condition is caused by vascular cell proliferation, intimal and medial hypertrophy, inflammation, and fibrosis. The sympathetic nervous system and renin-angiotensin-aldosterone system are involved in PAH pathogenesis [1, 2].

Several approaches to decrease sympathetic nervous activity in subjects with PAH have been reported, including transcatheter or surgical pulmonary artery denervation (PADN) [3], direct vagus nerve stimulation, and renal denervation (RDN) in experimental settings [4, 5].

Although the effects of RDN in earlier studies on systemic hypertension have been questioned, the results of recent studies seem more optimistic [6, 7]. Thus, RDN may currently be considered for resistant hypertension, but other pleiotropic effects related to modulation of sympathetic nervous activity have been reported. These include left ventricle hypertrophy reversal, renal function improvement, glycemic profile improvement, and attenuation of right ventricle (RV) remodeling in chronic PAH [8]. However, data on the effects of RDN on pulmonary hemodynamics are lacking.

Theoretically, the course of pulmonary hypertension may be aggravated by the activation of the efferent sympathetic bundles, leading to vasoconstriction, sodium retention, and release of renin from juxtaglomerular cells, and promoting the production of angiotensin II and aldosterone. Thus, deactivation of the afferent sympathetic bundles of the renal nerves may modulate the overall sympathetic nervous system activity and have positive hemodynamic effects in PAH.

RDN in induced PAH using dehydromonocrotaline in dogs has been previously described [5]. A few preclinical studies in small animals have evaluated the RV remodeling and pulmonary vascular effects of RDN [9, 10]. However, in these experimental studies, nonspecialized tools were used to perform renal denervation, which could affect the effectiveness of the procedure.

In a previous pilot experimental study, a decrease in pulmonary artery pressure (PAP) and pulmonary artery resistance has been detected in normotensive swine following extensive renal artery (RA) ablation [11]. Whether these effects are reproducible in induced PAH remains unknown.

Among several PAH models, intravenous thromboxane A2 (TXA2) infusion has been tested for the evaluation of acute hemodynamic changes after PADN [12]. Previous reports have described an escalating dosage protocol for reproducible PAH modeling [13]. Therefore, because of the rapid inducibility, stable PAP elevation, and complete reversibility after TXA2 withdrawal, we have chosen this model for the current experiment.

This study is aimed at assessing the effects of RDN on systemic and pulmonary hemodynamics in a swine model of TXA2-induced PAH.

## 2. Methods

### 2.1. Experimental Animals

This was a randomized experimental study with a sham procedure to evaluate acute hemodynamic changes after extended RDN. Ten normotensive breed Landrace swine (mean body weight 42.7 ± 4.0 kg, age 3 months) were included in the experiment. The Institutional Animal Care and Use Committee reviewed and approved all procedures and protocols for this study.

### 2.2. Intraoperative Procedures

All procedures were performed under general anesthesia. Sedation of the animals was performed using an intramuscular injection of 1.5 mL Zoletil 100 (Virbac, Carros, France), and the outer ear vein was cannulated for drug infusion. Then, intubation was performed using mechanical ventilation. Anesthesia was maintained by ventilation with 1% isoflurane (Baxter Healthcare Corp., Puerto Rico) using an anesthesia machine (WATO EX-35; Shenzhen Mindray Bio-Medical Electronics Co., Ltd, China) with the following parameters: FiO_2_ 0.3, tidal volume 10 mL/kg, and peak end-expiratory pressure 6 cmH_2_O. Vascular access was obtained through the right femoral artery and vein using the Seldinger technique. An 8F multipurpose sheath (Preface®, Biosense Webster, CA, USA) was placed into the right femoral artery, and 7F and 6F sheaths (AVANTI®+, Cordis, FL, USA) were inserted into the right femoral vein. Immediately after obtaining vascular access, heparin was delivered to reach an activated clotting time (ACT) over 300 s; ACT was assessed every 30 min, and additional intravenous heparin was administered, if necessary.

### 2.3. Hemodynamic Monitoring

Continuous invasive blood pressure monitoring was performed during the procedure. Measurement of hemodynamic parameters was described in detail earlier [11].

The baseline hemodynamic parameters were considered after a 20 min waiting period following vascular sheath placement. A Swan-Ganz catheter (6F Corodyn™P2, BRAUN, Bethlehem, Germany) was used for right heart catheterization, and the following parameters were assessed: heart rate (HR), invasive blood pressure (BP), PAP, pulmonary artery wedge pressure (PWP), RV pressure, and right atrial pressure (RAP). Arterial blood samples were obtained from the abdominal aorta through a multipurpose sheath, and venous blood samples were obtained from the PA. Blood tests were performed using a portable analyzer (i-STAT; Abbott Laboratories, IL, USA). Cardiac output (CO) was calculated using Fick's equation. Pulmonary vascular resistance (PVR) and systemic vascular resistance (SVR) were calculated using established formulas as previously reported [11].

**Figure 1:**
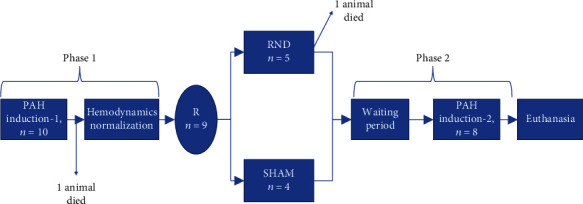
Study flowchart. PAH: pulmonary arterial hypertension; R: randomization; RDN: renal denervation; SHAM: sham procedure.

During the induction of PAH, a constant recording of systemic BP and PAP was performed. After the first PAH induction, a time interval of 20–30 min was allowed until the baseline PA pressure was completely restored. Then, the RDN or sham procedure was performed according to the allocation group. A second PAH induction protocol was then performed after at least 30 min. The study flowchart is shown in Figure 1.

### 2.4. PAH Induction Using the Synthetic Analogue of Thromboxane A2

PAH induction was performed twice, at baseline and following RDN or the sham procedure, depending on the allocation group, using a synthetic analogue of TXA2 (U46619; 10 mg/mL; Tocris Bioscience, MN, USA). During induction, the mean PA pressure was targeted at 40 mmHg. Continuous administration of TXA2 was gradually increased every 5 min according to a previously established protocol [13]. Electrocardiography (ECG) and both systemic pressure and PAP were continuously recorded. After the target PA pressure level was achieved, PWCP, PA pressure, RV, and RA pressures were measured and recorded; CO, SVR, and PVR were calculated before induction, at the target mean PA pressure, and after spontaneous reversal of mean PA pressure to baseline.

### 2.5. Group Allocation

After the first PAH induction and spontaneous normalization of hemodynamic parameters, experimental animals were randomly allocated to group #1 (*n* = 6), bilateral RDN with repeatedly induced PAH, or group #2 (*n* = 4), a sham procedure. Blocked randomization was performed using an online randomization service (https://www.sealedenvelope.com/simple-randomiser/v1/lists).

### 2.6. Renal Artery Characterization and Denervation Procedure

Selective angiography of the right and left RAs was performed using 20 mL of contrast medium (Optiray 300, Guerbet, France). After the first PAH induction, PA pressure restoration to a baseline level, and group allocation, isosorbide dinitrate 0.1% (Isoket, EVER Pharma, Germany) was delivered selectively into both RAs through a vascular sheath at a dose of 50 *μ*g. Then, a radiofrequency (RF) ablation balloon catheter (Vessix™, Boston Scientific, Natick, MA, USA) was placed into each RA via a 0.014 in ×185 cm guidewire (PT2, Boston Scientific).

In the RDN group, ablation was performed bilaterally in all available RA branches and segments. Each RF application was set at 30 s in duration, temperature 65–68°C, and automatically adjusted power of 0.5–1.0 W.

In the sham group, the procedure was performed using the same protocol, including angiography, nitrate injection, and the number and duration of balloon inflation. However, no RF energy was delivered. After RDN or balloon inflation, a waiting period of 30 min was considered, and the PAH induction procedure was repeated.

Hemodynamic parameters were compared between phases 1 and 2 of the study in each group and between the groups.

### 2.7. Autopsy Study

At the end of the experiment, all animals were euthanized using a lethal dose of intravenous potassium chloride solution (OZON Pharm, Samara, Russian Federation). After the onset of biological death, the kidneys and abdominal aorta with the RAs were excised as a single unit and fixed in a solution of 10% buffered formalin for further gross examination. The RAs were cut longitudinally from the ostia to the renal parenchyma and evaluated macroscopically. Visually detected lesions were calculated and excised within normal tissue (3–5 mm borders around a lesion) through all artery wall layers (intima, media, adventitia, and perivascular fat tissue) and prepared in paraffin blocks. Then, the items were sampled with a 1 mm step into slices 3 *μ*m thick and stained with hematoxylin and eosin.

Unaltered and damaged nerves were calculated for each sample, the median number of total nerve fibers was calculated per lesion, and the percentage of damaged nerves was calculated per lesion. Nerve damage was considered when a nerve fiber was affected by a lesion, compressed by a hematoma, or totally destroyed.

### 2.8. Outcome Measures

The primary endpoint was a change in TXA2 dose required to achieve a mean PAP of 40 mmHg after RDN in comparison with the sham group. The secondary endpoint was a change in the time required for TXA2 infusion until the target mean PAP was reached following RDN.

### 2.9. Statistical Analysis

Continuous variables are presented as mean ± standard deviation or median with interquartile ranges, and categorical variables are presented as percentages. Group comparisons were performed using the Mann-Whitney *U*-test. Correlations were analyzed using Pearson's test. A two-sided *P* value < 0.05 was considered statistically significant. The analysis was performed using Statistica 12.0 (StatSoft Inc., Tulsa, OK, USA).

## 3. Results

Two out of ten animals died during the procedure and were excluded from the analysis. One animal died of acute right ventricular failure with a decrease in systemic BP and ST-segment depression on the ECG during the first PAH induction protocol. The other death occurred 5 min after renal denervation: complete atrioventricular block developed with further asystole, despite temporary RV pacing, and the animal died of electromechanical dissociation. In both cases, resuscitation measures were unsuccessful. In the first case, autopsy revealed a massive pulmonary embolism. Therefore, data analysis was performed on eight animals (four in each group).

The total number of RF ablation points in the RDN group was 87 (21.7 ± 1.7 per animal); the range of ablation temperature was 67.1–68.5°C. The median number of balloon inflations inside the RAs was 4 [4; 6] per animal in either group.

### 3.1. Hemodynamic Parameters before and after Renal Denervation

The target PAP was achieved in all eight animals before and after the RDN and sham procedures. The hemodynamic parameters in the RDN and sham groups are shown in Table 1.

At PAH induction before and after RDN (phase 1 and phase 2), a statistically insignificant decline in the mean level of BP was noted (90.25 ± 28.1 vs. 74 ± 18.7 mmHg, *P* = 0.28). Similarly, the level of systemic vascular resistance (SVR) showed a trend towards a lower mean value (2433.7 ± 1176.7 vs. 1995.3 ± 494.3 *dyn*∗sec∗*cm*^−5^) after RDN at PAH induction, but the difference was not statistically significant (*P* = 0.46). There was also no statistically significant change in hemodynamic parameters in the sham group before and after the procedure. There was no statistically significant difference in the doses of TXA2 between the groups, either at the initial administration or during PAH induction following the procedure. Within each group, TXA2 doses for the first and second PAH induction were statistically equivalent (Table 2).

### 3.2. Renal Artery Wall and Nerve Lesions

**Figure 2:**
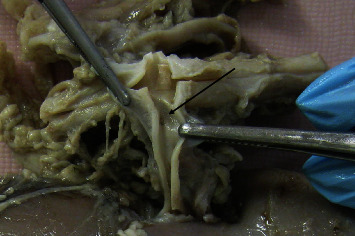
Pinpoint-like dissection in renal artery trunk. Findings in swine #3.

**Figure 3:**
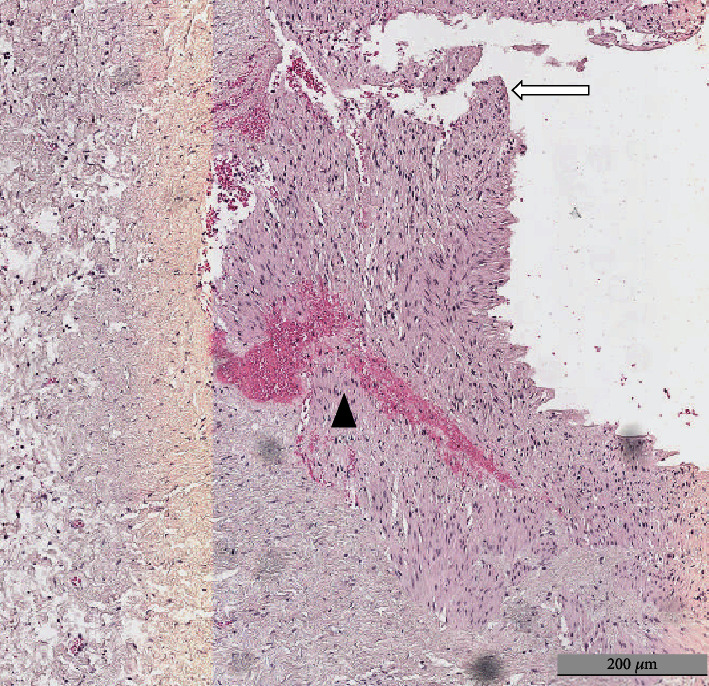
Microscopic evaluation of renal artery wall dissection (white arrow) and hemorrhagic impregnation (black arrow) after radiofrequency ablation. Findings in swine #3. Hematoxylin-eosin staining, 100x.

Gross anatomical evaluation of the RA showed the presence of artery dissections in both groups of animals, but the number of dissections was higher in the RDN group than in the sham group: 17 in total (4 [3; 4] per animal) vs. 3 in total (1 [1; 1] per animal) (*P* < 0.05) (Table 3). The shape of artery dissections was different between groups: pinpoint-like dissections in the sham group and larger dissections (length ranging from 2 to 5 mm) in the RDN group. An example of a macroscopically visible dissection after RDN is presented in Figure 2, while an example of microscopical examination of a RA wall dissection is shown in Figure 3.

**Figure 4:**
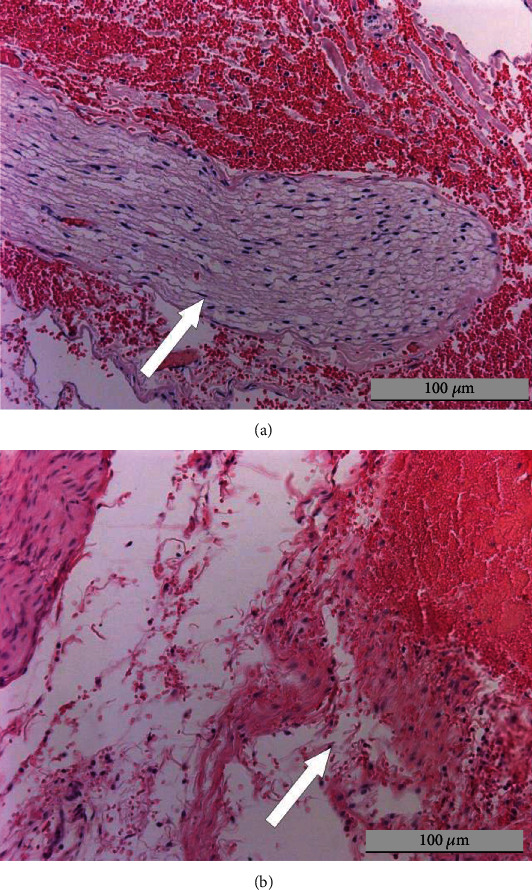
Microscopic evaluation of nerve damage. The arrows indicate an intact nerve fiber (a) in the sham group and a destroyed nerve fiber in the RDN group (b). Findings in swine #8 and #4. Hematoxylin-eosin staining, 200x.

Nerve damage was detected in both animal groups, with a significant prevalence in the RDN group: 34% vs. 3% of nerve fibers visualized by microscopy (Table 3 and Figure 4).

No blood clot was detected in any RA.

## 4. Discussion

In our experimental study, the primary outcome was not achieved; no change in the dose of TXA2 required for the increase of mean PAP up to 40 mmHg was observed after RDN. The secondary endpoint, the mean time of TXA2 infusion until the target PAP, showed no difference between the RDN and sham groups. Therefore, the main finding of this study is that RDN does not significantly influence hemodynamic parameters in TXA2-induced acute PAH. Although there was a trend toward a lower systemic BP after RDN at repeated induction of PAH, the difference was not statistically significant.

The long-term clinical effects of RDN on BP have been widely demonstrated, and the major results of RDN are prominent several weeks following the procedure [14, 15]. The acute impact of RDN on BP has been described in clinical and experimental studies and is mainly explained by the change in sympathetic nervous tone after ablation of the nerves around the RAs [16].

Our theoretical suggestion that RDN resulting in an overall sympatholytic effect might influence the inducibility of PAH in an animal model has not been proven in this experimental study. This may be explained by the following two assumptions. First, the TXA2 model of PAH induction is not suitable for our analysis because TXA2 is a direct vasoconstrictor and its effect cannot be attenuated by autonomic nervous tone. Second, the pathogenesis of acute PAH may not be related to sympathetic activity in animal models. The design of our study did not include remote assessment of RA denervation on pulmonary hemodynamics, and the chronic effects of the procedure on PAH inducibility should be evaluated in further studies.

In a recent report by our group, RDN in normotensive pigs led to rapid and significant changes in systemic BP and PVR [11]. These results were not confirmed in the present study. One of the potential reasons for this divergent finding is the possible prolonged effect of TXA2 on hemodynamic parameters due to peripheral vasoconstriction. Indeed, the initial administration of TXA2 may trigger compensatory regulation mechanisms of pulmonary circulation. Therefore, the second PAH induction was not associated with a lower dose of TXA2.

In one animal, a lethal massive pulmonary embolism possibly associated with TXA2 infusion was detected. The thrombogenic effect of TXA2 despite adequate anticoagulation might be responsible for this complication, as shown in a previous report [13].

In our study, all procedures were performed under the same conditions, namely, all animals were of approximately the same age and similar morphometric indicators, and general anesthesia with intubation was performed. The only difference between the groups was the number of RF applications.

Damage to the vascular wall of the RA was detected in all animals in the RDN group and in two animals in the sham group, suggesting that balloon inflation was performed adequately in both groups. These results confirm the previous results obtained in normotensive animals, where RA wall damage has been detected in RDN and sham-operated animals [11]. Here, we used the same ablation catheter, and the characteristics of artery wall lesions are very similar to previous findings. It should be noted that control angiography after RDN showed no changes in the contours of the vessels. Therefore, microtrauma to the RA wall is not seen on conventional angiography but can be found on macroscopic and microscopic artery evaluations. In contrast, perivascular nerve damage was detected in all the animals from the RDN group. Although not all nerve fibers were affected by ablation, the percentage of damaged tissue was similar to that in previous reports [17], histologically confirming that denervation was performed. Moreover, when distal ablation is performed, as in our study, more sympathetic denervation is seen as a decrease in norepinephrine spillover [18].

Previous studies in rodents have shown positive effects of renal denervation on monocrotaline-induced PAH. Thus, Liu et al. have found that earlier RDN treatment significantly decreases sympathetic nervous activity renin-angiotensin-aldosterone system (RAAS) activation and significantly improved the survival rate of experimental animals and attenuated cardiopulmonary fibrosis [19]. Similarly, another study by da Silva Goncalves et al. that have used monocrotaline and sugen 5416 for PAH induction reported that surgical RDN delayed PAH progression, reduced RV afterload and pulmonary vascular remodeling, and reduced diastolic stiffness, hypertrophy, and fibrosis of the RV. These beneficial effects have been linked to RAAS suppression [9]. In a canine model of PAH induced by dehydromonocrotaline injection, RDN attenuated pulmonary vascular remodeling and decreased pulmonary arterial pressure, as reported by Qingyan et al. [10]. We suggest that the difference in the results of our study from those demonstrating positive effects of RDN on PAH is mainly explained by chronic PAH models and the difference in RA innervation among species [20, 21].

In our study, 34% of nerves were found affected in the acute period following ablation, and one might find this a low damage incidence. Thus, in a study by Cohen-Mazor et al. where a very similar ablation protocol has been used, necrotic changes at 7 days following the procedure were detected in 29 and 44% of nerves following single-ablation and full-artery length ablation, correspondingly. The additional damage of 45 and 59% of nerves was characterized as “degenerative” and “chronic/reactive” reflecting delayed changes. Considering the difference in time of necropsy with our study, we speculate that the rate of nerve damage should be comparable. However, the analysis of nerve damage was different and certain diversity in local results of ablation may present. Therefore, there is a potential possibility that the lack of a BP drop or hemodynamic effects might be related to insufficient denervation rather than a mechanistic finding [22].

### 4.1. Implications for Future Studies

Since our neutral results of RDN on the inducibility of reversal of PAH using TXA2 and positive previous studies on chronic PAH models, we suggest that TXA2 might not be fully suitable for neuromodulation interventions for PAH and other models should be preferred. Indeed, acute hemodynamic changes following RDN detected in normotensive pigs were not reproduced in the TXA2-induced PAH model [11]. Another suggestion is that the delayed effects of RDN should be evaluated for possible hemodynamic changes, since perivascular nerve fiber damage develops over time following ablation.

### 4.2. Study Limitations

We evaluated the acute cardiovascular effects of RDN and did not assess its long-term effects.

Another limitation of this study is the small sample size of animals on which experimental studies were conducted. Thus, this study is exploratory and shows the effects of RDN on the inducibility of acute PAH in animals in an experimental operating room. However, the use of a sham group in the course of research makes the conclusions of the experimental work more significant.

Our protocol did not include high-frequency electrical stimulation of the RAs before and after RF ablation in the RDN group. Given the inconsistent results of this approach in the identification of successful denervation from previous studies, including our recent report [11], we decided to omit this step in the present study.

Additionally, our study protocol did not include the analysis of norepinephrine spillover before and after RDN. However, previous studies reported a significant drop in its level after ablation in the distal and proximal RA branches, as we implemented in the current study [23].

In a previous report of Tzafriri et al., where a novel spiral multielectrode ablation system was implemented, a systematic correlation of 7d histological nerve effects with norepinephrine reduction determined >46% affected nerves as the threshold for statistically significant norepinephrine reduction [24]. While the time point used by the authors and the methodology are different from our study, the above-mentioned difference in nerve damage weakens our mainly neutral results on the effects of RDN on acute hemodynamic changes.

## 5. Conclusions

According to the results of our experimental study, RDN does not lead to significant hemodynamic changes in an animal model of acute TXA2-induced PAH. The potential chronic effects of RDN on pulmonary hemodynamics require further research.

## Figures and Tables

**Table 1:** Hemodynamic parameters before and after the RDN and sham procedures.

	RDN group, *n* = 4				Sham group, *n* = 4			
	Normal hemodynamics		TXA2 infusion		Normal hemodynamics		TXA2 infusion	
	Before RDN, phase 1^∗^	After RDN, phase 2^∗∗^	Before RDN, phase 1^∗^	After RDN, phase 2^∗∗^	Before sham, phase 1^†^	After sham, phase 2^††^	Before sham, phase 1^†^	After sham, phase 2^††^
HR, min^−1^	82.5 ± 11.6	90 ± 18.4	101.7 ± 21.1	105 ± 18.8	98.75 ± 9.1	102.25 ± 1.9	110.25 ± 8.2	115.75 ± 14.9
sBP, mmHg	96 ± 7.6	103.5 ± 18.2	109.75 ± 94.25	94.25 ± 27.8	99.75 ± 13.6	105.75 ± 6.9	103 ± 6.9	104 ± 36.5
dBP, mmHg	64.5 ± 2.1	65 ± 10.5	80 ± 26.3	63.75 ± 14.2	53.25 ± 13.7	59.25 ± 8.1	61.5 ± 3.4	62.25 ± 24.3
mBP, mmHg	74.75 ± 4.1	78 ± 12.6	90.25 ± 28.1	74 ± 18.7	68.75 ± 14.3	73 ± 8.8	75.5 ± 4.2	76 ± 27.9
sPAP, mmHg	18.5 ± 3.3	20 ± 2.6	48.25 ± 9.6	47.5 ± 6.2	17.25 ± 2.6	15.75 ± 2.9	44.25 ± 2.5	42.75 ± 0.9
dPAP, mmHg	11.5 ± 3.1	11.5 ± 3	34.75 ± 8.8	34.5 ± 5.7	12.75 ± 4.3	12.5 ± 3	39 ± 0.8	38 ± 0.8
mPAP, mmHg	14 ± 2.9	14.5 ± 3	40.5 ± 7.3	39 ± 5.3	14.5 ± 4.1	13.5 ± 3	41.25 ± 1.25	39.75 ± 0.5
PWP, mmHg	6 ± 2.4	4.25 ± 3	8.25 ± 1	8.25 ± 2.7	4.75 ± 1.7	4.6 ± 0.6	7 ± 1.8	6.75 ± 0.9
RAP, mmHg	5.5 ± 2.6	2.75 ± 1.7	7.75 ± 2.6	5.25 ± 2.1	4 ± 3.1	4.3 ± 1.5	6.25 ± 2.6	5.25 ± 2.2
SVR, dyn∗sec∗cm^−5^	1424.1 ± 559.8	1439.6 ± 114.8	2433.7 ± 1176.7	1995.3 ± 494.3	1576.54 ± 328.9	1657.1 ± 356.1	2272.6 ± 917.6	2373.7 ± 692.8
PVR, dyn∗sec∗cm^−5^	161.9 ± 51.3	199.1 ± 47.7	1086.2 ± 361.8	978.8 ± 310.1	1086.2 ± 361.8	978.8 ± 310.1	1121.2 ± 456.3	1228.9 ± 524.3

Data are presented as mean ± standard deviation. Within each group, a significant change in all hemodynamic parameters was noted at PAH induction. ^∗^*P* < 0.05 between normal hemodynamics and PAH before RDN. ^∗∗^*P* < 0.05 between normal hemodynamics and PAH after RDN. ^†^*P* < 0.05 between normal hemodynamics and PAH before sham procedure. ^††^*P* < 0.05 between normal hemodynamics and PAH after sham procedure. *P* > 0.05 between the RDN and sham groups for all parameters, when compared with normal hemodynamics. *P* > 0.05 between the RDN and sham groups for all parameters, when compared at PAH induction. HR: heart rate; sBP: systolic blood pressure; dBP: diastolic blood pressure; mBP: medium blood pressure; sPAP: systolic pulmonary artery pressure; dPAP: diastolic pulmonary artery pressure; mPAP: medium pulmonary artery pressure; sRV: systolic right ventricle pressure; dRV: diastolic right ventricle pressure; mRV: medium right ventricle pressure; PWP: pulmonary wedge pressure; RAP: right atrial pressure; SVR: systemic vascular resistance; PVR: pulmonary vascular resistance; CO: cardiac output.

**Table 2:** Doses and duration of TXA2 infusion until target mean PAP.

	RDN group (*N* = 4)		*P*	Sham group (*N* = 4)		*P*	*P* value between RDN and sham groups before denervation or balloon inflation	*P* value between RDN and sham groups after denervation or balloon inflation
	Before RDN	After RDN		Before sham	After sham			
TXA2 dose, mcg/min (mean ± SD; median [IQR])	0.1 ± 0.02 0.11 [0.08; 0.12]	0.09 ± 0.01 0.1 [0.08; 0.1]	0.58	0.12 ± 0.03 0.12 [0.09; 0.13]	0.11 ± 0.04 0.875 [0.07; 0.13]	0.59	0.56	1.0
TXA2 dose, mcg (mean ± SD; median [IQR])	23.9 ± 5.1 25.1 [20.3; 27.5]	21.5 ± 2.6 22.2 [19.9; 23.2]	0.46	22.6 ± 6.2 22.84 [18.4; 26.8]	20.5 ± 8.4 16.95 [15.2; 25.9]	0.59	0.66	0.47
Time until mPAP reaches 40 mmHg (mean ± SD; median [IQR])	17.5 ± 2.9 17.5 [15; 20]	17.2 ± 3.2 17.5 [14.5; 20]	0.79	16.7 ± 6.7 19.0 [12.5; 21]	15.7 ± 7.1 13.5 [11; 20.5]	0.71	0.88	0.56

IQR: interquartile range; mPAP: medium pulmonary artery pressure; PAP: pulmonary artery pressure; RDN: renal denervation; SD: standard deviation; TXA2: thromboxane A2.

**Table 3:** Characteristics of renal artery wall lesions and nerve damage per group.

Group	Number of RF lesions	Median number of artery wall dissections per animal	Median number of nerves per mm^2^	% of damaged nerve fibers, total percentage per group
RDN, *n* = 4	21.7 ± 1.7	4 [3; 4]	7 [7; 9]	34%
Sham, *n* = 4	0	1 [1; 1]	7.5 [6.8; 9]	3%
*P*	—	NS	NS	<0.01

NS: nonsignificant; RDN: renal denervation; RF: radiofrequency.

## Data Availability

The data used to support the findings of this study are available from the corresponding author upon reasonable request.
